# Using Transcriptome Analysis to Screen for Key Genes and Pathways Related to Cytoplasmic Male Sterility in Cotton (*Gossypium hirsutum* L.)

**DOI:** 10.3390/ijms20205120

**Published:** 2019-10-16

**Authors:** Yuqing Li, Tengfei Qin, Chunyan Wei, Jialiang Sun, Tao Dong, Ruiyang Zhou, Quanjia Chen, Qinglian Wang

**Affiliations:** 1 College of Agriculture, Xinjiang Agricultural University, Urumqi 830000, China; lyq120327@163.com; 2 School of Life Science and Technology, Henan Institute of Science and Technology/Collaborative Innovation Center of Modern Biological Breeding of Henan Province/Henan Key Laboratory Molecular Ecology and Germplasm Innovation of Cotton and Wheat, Xinxiang 453003, China; qintengfeisam@163.com (T.Q.); weichunyan688@163.com (C.W.); sjl5845@126.com (J.S.); dongtao985@163.com (T.D.); 3 Key Laboratory of Plant Genetics and Breeding, College of Agriculture, Guangxi University, Nanning 530006, China; ruiyangzh@gmail.com

**Keywords:** cotton, cytoplasmic male sterility, transcriptome, DEGs

## Abstract

Cotton (*Gossypium hirsutum* L.) is one of the most important cash crops worldwide. Cytoplasmic male sterility (CMS) is an excellent breeding system for exploitation of heterosis, which has great potential to increase crop yields. To understand the molecular mechanism of CMS in cotton, we compared transcriptome, cytomorphological, physiological and bioinformatics data between the CMS line C2P5A and its maintainer line C2P5B. By using high-throughput sequencing technology, 178,166 transcripts were assembled and 2013 differentially expression genes (DEGs) were identified at three different stages of C2P5A anther development. In this study, we identified DEGs associated with reactive oxygen species (ROS), peroxisomes, aldehyde dehydrogenases (ALDH), cytochrome oxidase subunit VI, and cytochrome P450, and DEGs associated with tapetum development, Jojoba acyl-CoA reductase-related male sterility protein, basic helix-loop-helix (bHLH) and MYB transcription factors. The abnormal expression of one of these genes may be responsible for the CMS C2P5A line. In gene ontology (GO) annotation and Kyoto Encyclopedia of Genes and Genomes (KEGG) pathway enrichment, DEGs were mainly related to carbohydrate metabolism, amino acid metabolism, transport and catabolism, and signal transduction. Carbohydrate metabolism provides energy for anther development, starch and sucrose metabolism, fatty acid biosynthesis and metabolism and ascorbate and aldarate metabolism. These results showed that numerous genes and multiple complex metabolic pathways regulate cotton anther development. Weighted correlation network analysis (WGCNA) indicated that three modules, ‘turquoise,’ ‘blue,’ and ‘green,’ were specific for the CMS C2P5A line. The ‘turquoise’ and ‘blue’ modules were mainly related to carbohydrate metabolism, amino acid metabolism, energy metabolism, peroxisomes, pyruvate metabolism as well as fatty acid degradation. The ‘green’ module was mainly related to energy metabolism, carbon metabolism, translation, and lipid metabolism. RNA-sequencing and WGCNA polymerization modules were screened for key genes and pathways related to CMS in cotton. This study presents a new perspective for further research into the metabolic pathways of pollen abortion in the CMS C2P5A line and also provides a theoretical basis for its breeding and production.

## 1. Introduction

Cotton (*Gossypium hirsutum* L.) is one of the most important cash crops in the world and a renewable source of natural fiber [1]. At present, there are four cultivated varieties of cotton, diploid Asian cotton (*G. arboreum*), African cotton (*G. raimondii*), upland cotton (*G. hirsutum*), and island cotton (*G. barbadense*). Upland cotton accounts for more than 90% of commercial cotton production worldwide because of its excellent characteristics such as high fiber yields, strong adaptability, high resilience, and good quality [2,3,4].

At present, heterosis is the most effective way of high yield breeding. It makes a great contribution to the increase of agricultural yields because hybrid F1 progenies possess merit traits such as increased adaptability, uniformity, and stronger stress tolerance [5,6]. Heterosis systems have been used to increase yields in several crop species such as wheat [7,8], rice [9], cereals [7,10], soybeans [11], barley [7,12], cotton [13], and rapeseed [14].

In male sterility (MS), the pistil of the bisexual flower plant develops normally, but the stamens fail to produce a dynamic male gamete, therefore, plants can be fertilized by external pollination. MS is widespread in flowering plants as a tool to create new hybrids for breeders, which can greatly reduce labor, costs, and improve production efficiency [15]. According to gene composition, MS includes genic male sterility (GMS) and cytoplasmic male sterility (CMS) [16].

GMS is a two-line breeding system, according to morphological characteristics, the fertile plants and fake hybrids have to be removed, which results in increased labor intensity and operation difficulty. CMS is a three-line breeding system, which can eliminate the need for hand emasculation. CMS has been found in more than 150 species [17], including 7% of the gynodioecy angiosperms [18]. CMS confers an excellent system for hybrid breeding system, especially for monoecious crops, which exploits crop heterosis and produces large-scale commercial F1 hybrid seeds [9,11]. Bohr et al. believed that hybridization or heterosis is the most promising method to solve the challenge of increasing crop yields [11].

Anther development is a complex process involving numerous genomes and multiple metabolic pathways in cells. The carbohydrate metabolism pathway provides energy and carbohydrates for anther development [19,20,21,22]. Lipid transport and fatty acid metabolism pathway play crucial roles in signal transduction and sporopollenin synthesis [23,24,25]. Ascorbate and glutathione metabolism are involved in anther cell development and meiosis [19,26,27,28]. The flavonoid biosynthesis pathway is involved in the process of forming the pollen coat [19,29,30].

Numerous genes related to anther development have been identified in model plants like Arabidopsis (*Arabidopsis thaliana*) and rice (*Oryza sativa*). *SPL*/*NZZ*, *EMS1*/*EXS*, *TPD1*, *SERK1*, and *SERK2* regulate the fate of somatic, germ cells and the differentiation of the anther wall during early anther development [31,32,33,34,35]. *BAM1*/*BAM2* are important regulatory factors for early anther development [36,37]. *MPK3*/*MPK6* regulate anther chamber formation [38], *DYT1* and *22DYT1* are downstream transcription factors [39,40]. *RPK2* [41], and *TDR* [42,43,44] regulate and control the degradation of tapetum cells and microspore development. *Defective tapetum Cell Death 1 (DTC1)* controls tapetum degeneration by regulating ROS dynamics in rice [44], *MIL1 and MIL2* are required for anther and microspore development [45,46]. *UDT1, TDR,* and *TIP2* encoding bHLH transcription factors regulate stamen differentiation and anther development [44,47,48]. *PTC1* [49], *EAT1* [50], *API5* [51], and *TIP2/bHLH142* [44] regulate programmed tapetal cell death and pollen development. *WA352* gene interacts with *COX11*gene to cause tapetal programmed cell death (PCD) [52].

The *GhACS1* gene encodes an acyl-CoA synthetase which is essential for normal microsporogenesis in the early anther development of cotton [53]. *GhMYB24* encodes the MYB-like transcription factor that regulates the development of tapetum [54].

With regards to the relationship between male sterility and the homeostasis of reactive oxygen species (ROS), previous studies have shown that the accumulation of ROS is higher in male sterile plants, for instance, in rice [52,55], wheat [56], maize [57] and cotton [27,58], indicating that ROS accumulation may be closely related to the development and functional defects of tapetum [48,52,59].

Previous reports have shown that the formation of CMS mainly involves the mutation or molecular rearrangement of genes in the mitochondria or chloroplast genome [60,61]. Chen et al. believed that CMS is a complex character [62]. Mitochondrial genes that determine male sterility have been found in many plants, most of these genes are chimeric [63,64,65]. In total, 28 CMS genes have so far been identified in 13 crops [9].

Hua and his colleagues compared mitochondrial genome differences in cotton of the male sterile lines CMS-2074A, CMS-2074S, and maintainer and restorer lines, and identified four chimeric gene ORFs, *Aorf4, Aorf9, Aorf4-2,* and *Aorf28*, in the male sterile lines (CMS-2074A) and restorer lines with different transcription levels. The study revealed the cause for the formation of CMS in cotton [66].

To explore the CMS mechanism of abortion in cotton, the morphological, the physiological, and the molecular characteristics of the CMS line C2P5A and the maintainer line C2P5B were studied. Morphological changes and changes in the tapetum at different stages of anther development were analyzed, pollen viability was detected by triphenyltetrazolium chloride (TTC) staining and observed by scanning electron microscopy (SEM).

In this article, we used high-throughput sequencing technology and WGCNA polymerization modules to screen for key genes and pathways related to CMS in cotton by comparative transcriptome analysis. Besides, this study used quantitative Reverse-Transcription PCR (qRT-PCR) technology to verify the expression of genes related to fertility selected from RNA-seq. The results of this study will provide new data for further research of the metabolic pathway of cotton abortion in the CMS line C2P5A, as well as a theoretical basis for its use in breeding and seed production.

## 2. Results

### 2.1. Morphological Characteristics of the CMS Line C2P5A and the Maintainer Line C2P5B

**Figure 1:**
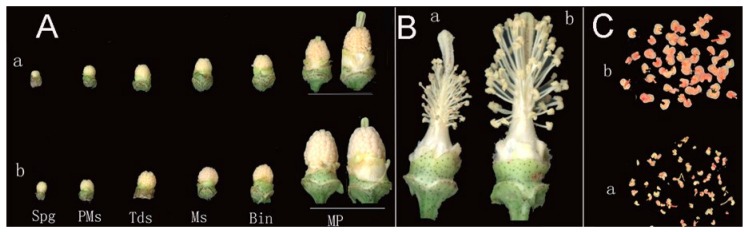
The morphological characteristics of flower buds at different development stages. a, cytoplasmic male sterile line C2P5A; b, maintainer line C2P5B. (**A**) Flower buds after removing sepals and petals. (**B**) Flower with sepals and petals removed. (**C**) Determination of pollen grain activity by TTC. Spg, sporogenous stage; Pms, pollen mother cell stage; Tds, tetrad stage; Ms, mononuclear stage; Bin, binucleate stage; Mps, mature pollen grain stage.

Comparing the morphology of CMS line C2P5A and maintainer line C2P5B revealed the abnormal development of flower buds and the mature pollen (MP) grain stage in the CMS line C2P5A, and the flower phenotype of C2P5A was small and wizened (Figure 1A).

The CMS line with exposed styles and without pollen have significantly shorter filaments than maintainer line (Figure 1B). Pollen grains of CMS line stained by 2% TTC were dry, shape and size were nonuniformity, mostly not colored or lightly colored, and activity was significantly weaker than that of the maintainer line (Figure 1C).

In this study, flower buds were selected from CMS line and maintainer line of anther development at three different stages, and 1.5% (*w*/*v*) acetocarmine staining and cross-section were used to confirm the developmental stage, pollen mother cell stage (3–4 mm), tetrad stage (4.1–5.0 mm), mononuclear (5.1–6.0 mm) development at three different stages, and flower bud length (length from nectary to bud apex, mm).

### 2.2. Microstructure of Anther at Different Developmental Stages of the CMS Line C2P5A

**Figure 2:**
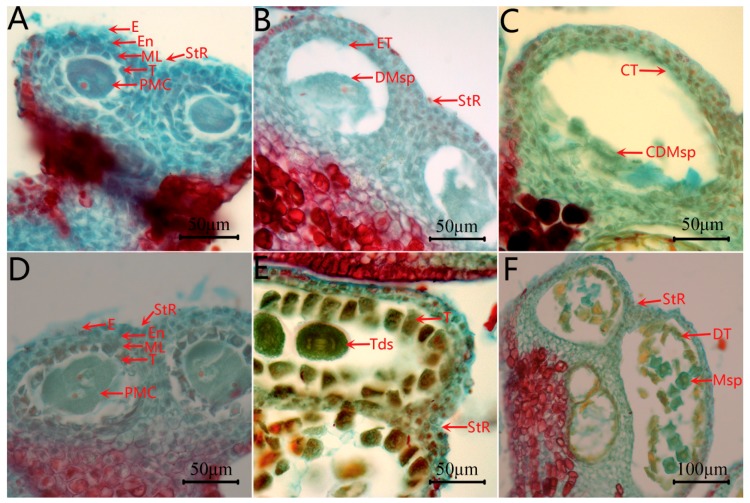
Phenotypic comparison between C2P5A and C2P5B at different development stages. (**A**–**C**) CMS line C2P5A; (**D**–**F**) maintainer line C2P5B. (**A**,**D**) Pollen mother cell stage (Pms); (**B**,**E**) Tetrad stage (Tds); (**C**,**F**) Mononuclear stage (Ms). PMC, pollen mother cell; E, epidermis; En, endothecium; ML, middle layer; Msp, microspores; T, tapetum; DT, degenerated tapetum; Tds, tetrads; Mn, mononuclear; DMsp, degenerated microspores; CDMsp, complete degenerated microspores; CT, compact tapetum; ET, entire tapetum; StR, stomium region.

When comparing the anther development of C2P5A with C2P5B by observing cross-sections, there was little difference between the two lines in pollen mother cell stages (Pms). Pollen mother cells were surrounded by four layers of cells, the innermost layer being the tapetum, followed by the middle layer, the endothecium, and the epidermal layer (Figure 2A,D). However, during the tetrad and mononuclear stages, there was a significant difference between the CMS line C2P5A and the maintainer line C2P5B (Figure 2B,C,E,F). The pollen mother cells of the CMS line C2P5A were degraded, the tapetum cells were atrophic, dense, and became a very thin layer (Figure 2B), and during the cells monocyte stage, no monocyte microspores were produced (Figure 2C).

### 2.3. Measurements of Physiological Indices in the CMS Line C2P5A

**Figure 3:**
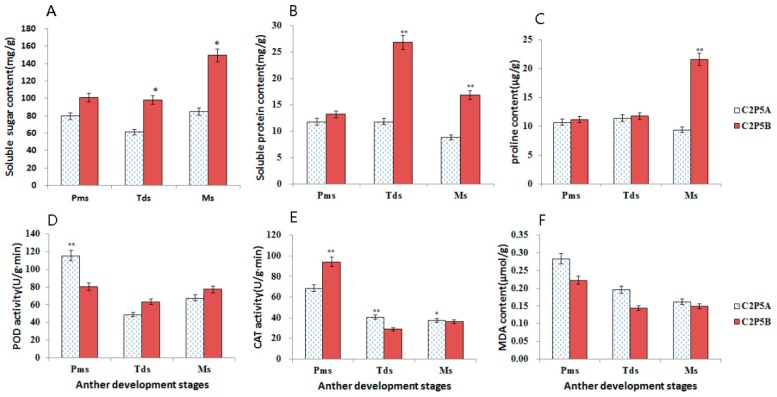
Determination of physiological indices of anthers at different developmental stages. **(A)** Determination of soluble sugar content. (**B**) Determination of soluble protein content. (**C**) Determination of proline content. (**D**) Determination of POD activity. (**E**) Determination of CAT activity. (**F**) Determination of MDA activity. PMs, pollen mother cell stage; Tds, tetrad stage; Ms, mononuclear stage; C2P5A, CMS line; C2P5B, maintainer line. The white columns indicate male sterile line C2P5A; red columns indicate the maintainer line C2P5B. The error bars represent SD (** *p* < 0.01, * *p* < 0.05), three biological replicates were performed.

Soluble sugar provides energy for anther development and synthesis. Soluble proteins include a variety of amino acids that protect cell development. At different stages of anther development, the soluble sugar and soluble protein content of the maintainer anthers was higher than that of the male sterile anthers. During anther development, significant differences could be observed in Tds and Ms between C2P5A and C2P5B (Figure 3A,B). Proline (Pro) is involved in the stress response in plants, but there was little difference in proline content of Pms and Tds during anther development in C2P5A and C2P5B. However, Pro in C2P5A was lower than in C2P5B at the Ms stage of anther development (Figure 3C).

Antioxidase peroxidase (POD) can eliminate the toxicity of hydrogen peroxide, phenols, amines, aldehydes, and benzene, whereas catalase (CAT) can remove hydrogen peroxide (H_2_O_2_) in the organism. At Pms stage in anther development, before anther abortion, POD and CAT activity increased correspondingly and reached significant levels at different stages in both lines. In the Pms stage, POD activity was significantly higher in the male sterile line than in the maintainer line (Figure 3D). However, CAT activity was significantly lower in C2P5A than C2P5B (Figure 3E). At Tds and Ms stage of anther development, the POD activity of male sterile line was lower than that of maintainer line (Figure 3D). However, the CAT activity was higher and reached a significant different level in the male sterile line (Figure 3E). Malondialdehyde (MDA) is an indicator of ROS in organisms, and the results showed that the MDA content in the male sterile line was higher than that in the maintainer line during all three anthers stages (Figure 3F).

### 2.4. Identification of Differentially Expressed Genes (DEGs)

**Figure 4:**
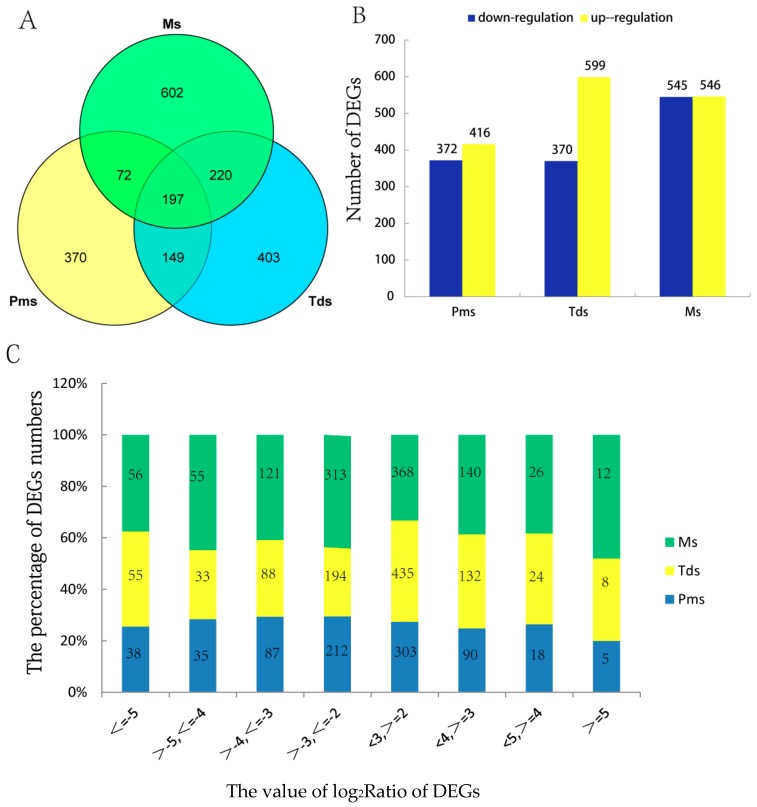
Analysis of DEGs in the male sterile line C2P5A compared to those of the maintainer line C2P5B from Pms to Ms stage of anther development. (**A**) Venn diagrams showing all DEGs shared among the three stages. (**B**) Number of DEGs that were up or down-regulated in the three development stages. (**C**) The distribution of the log_2_Ratio of DEGs in the C2P5A compaired to the C2P5B, Pms, Pollen mother cell stage; Tds, Tetrad stage; Ms, Mononuclear stage.

In this research, a total of 18 *Gossypium hirsutum* samples (three biological replications, three anther stages, two lines C2P5A and C2P5B) were sequenced using the Illumina HiSeq platform. A total of 178,166 transcripts were assembled, including 74,292 novel transcripts and 103,874 known transcripts. The number of total genes and new transcripts per sample were presented in Table 1. Clean reads (77743) were aligned to the reference sequence with Bowtie2 [67], and then the expression levels of genes and transcripts were calculated by RNA-Seq by Expectation Maximization (RSEM) [68] (Supplementary Table S1). Fragments per kilobase of transcripts per million mapped reads (FPKM) is the standard method to calculate gene expression levels by RSEM. Transcripts that were not entirely annotated to cotton were removed [69]. In this study, analysis of the difference between groups was conducted using the software DEGseq [70]. According to the threshold values: |log_2_Ratio| ≥ 2, readnum >3, *p* ≤ 0.001 and the criteria of FDR < 0.01 (Supplementary Table S2a). Overall, 788, 969, and 1091 differentially expressed genes (DEGs) were identified at the three stages (Pms, Tds, Ms), respectively, and 197 genes were shared among the three stages. A total of 2013 DEGs were identified (Figure 4A,B, Supplementary Table S2a–e). Analysis by log_2_Ratio found that the percentage of DEGs were mainly distributed from 2 to 3 and from −2 to −3 during Pms and Tds, and during Ms the percentage of DEGs was distributed widely (Figure 4C). The results showed that the male sterility had an effect on transcriptional changes in the anthers of C2P5A. At different stages of anther development, DEGs were up or down-regulated compared to C2P5B, indicating that a minimum number of DEGs occurred at the Pms stage of anther development rather than at the Tds and Ms stage of anther development. At Pms and Tds stage of anther development, more DEGs were found to be down-regulated than up-regulated. At Ms stage of anther development, almost an equal number of DEGs were up-regulated and down-regulated (Figure 4B). This may suggest that at Pms and Tds stage of anther development, the DEGs contain critical information related to male sterility.

### 2.5. Gene Ontology Annotation and Pathway Enrichment Analysis of DEGs

**Figure 5:**
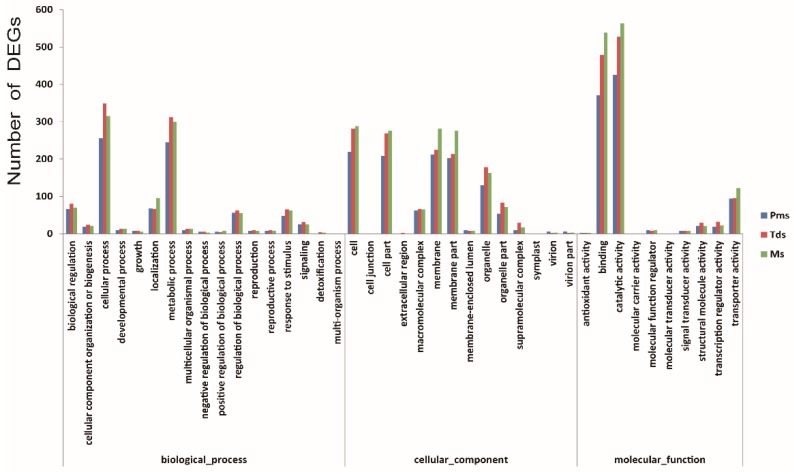
GO function enrichment of DEGs between the C2P5A and the C2P5B from the Pms to Ms stage of anther development. Pms, pollen mother cell stage; Tds, tetrad stage; Ms, mononuclear stage.

**Figure 6:**
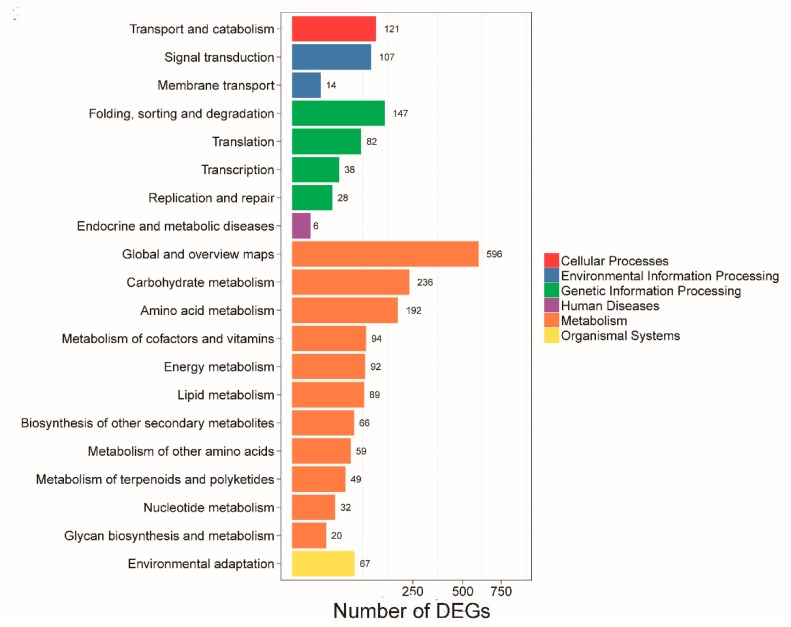
KEGG pathway enrichment analysis of 2,013 DEGs.

The results of gene ontology (GO) annotation and function enrichment of 2013 DEGs were distributed over 41 GO terms (Figure 5, Supplementary Table S3a). Molecular function, binding, and catalytic activity were related to the highest DEG number, followed by transporter activity. These results suggest that anther development in CMS may be associated with different genes. The Kyoto Encyclopedia of Genes and Genomes (KEGG) pathway enrichment was used to identify major biochemical and signal transduction pathways where the DEGs participated. In this experiment, pathway enrichment analysis showed mainly enrichment in 20 pathway categories including 125 KEGG pathways (Figure 6, Supplementary Table S3b). Global and overview maps, carbohydrate metabolism, folding, amino acid metabolism, sorting and degradation were the four most significant pathways, followed by transport and catabolism, signal transduction, metabolism of cofactors and vitamins, and energy metabolism. These results indicated that multiple complex metabolic pathways were involved in the anther development of cotton.

### 2.6. Correlation Network Analysis with WGCNA

**Figure 7:**
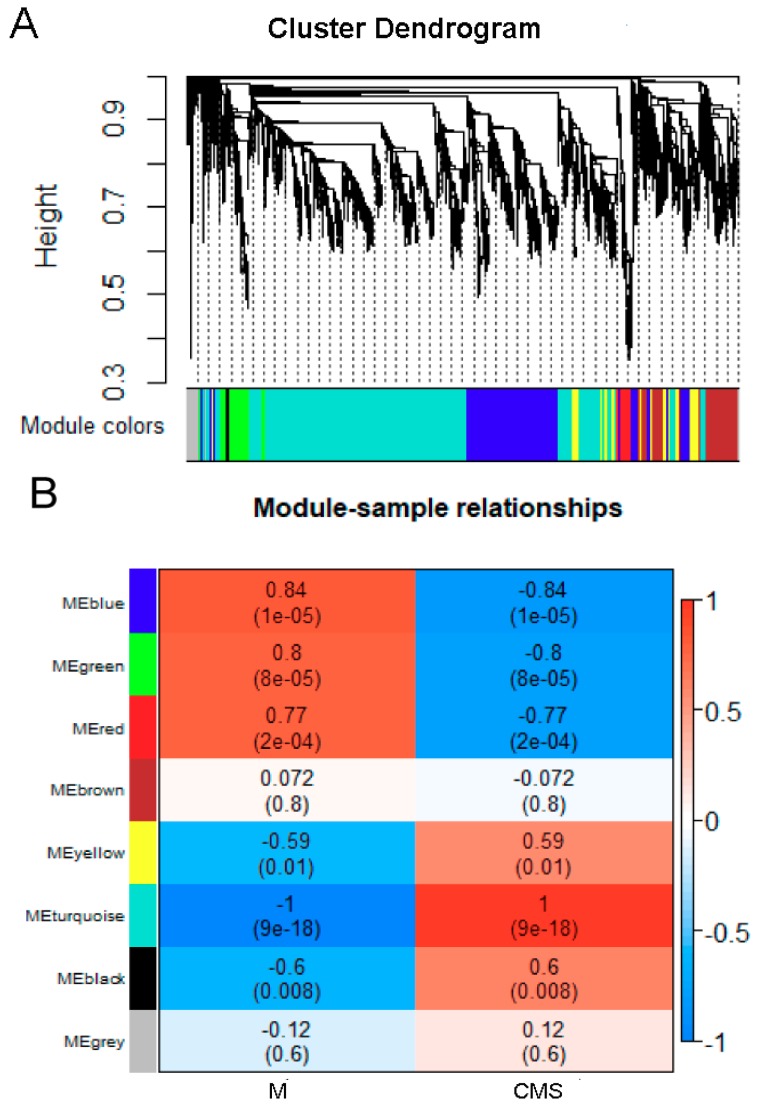
WGCNA analysis of CMS related genes. (**A**) Hierarchical clustering tree showing co-expression modules. In the tree, each leaf represents one gene. The major tree branches constitute seven modules, labeled by different colors. (**B**) Module-trait relationship. High correlation between a specific module and the sample is shown in dark red or dark blue. M, maintainer line C2P5B; CMS, cytoplasmic male sterile line C2P5A.

The weighted correlation network (WGCNA) is an alternative tool to explore target genes at a network-level, instead of individual genes [15,71,72]. In WGCNA, a module refers to a highly interconnected gene cluster, and within the same cluster the correlation coefficient is higher in the same tissue. To identify genes associated with CMS, seven modules were identified from the transcriptome data (Figure 7A).

Different samples were correlated with seven distinct modules, module ′turquoise′ (*r* = 1, *p* = 9 × 10^−18^), module ′blue′ (*r* = 0.84, *p* = 1 × 10^−5^), and module ′green′ (*r* = 0.8, *p* = 8 × 10^−5^) were highly correlated with the CMS line (Figure 7B).

The ‘turquoise’ module contained 3670 genes that were mainly related to genetic information processing, carbohydrate metabolism, amino acid metabolism, and energy metabolism (Supplementary Table S4a). The ‘blue’ module contained 1415 genes that were mainly involved in protein folding, sorting and degradation, amino acid metabolism, glycolysis/gluconeogenesis, peroxisomes, pyruvate metabolism, and fatty acid degradation (Supplementary Table S4b). The 359 genes in the ′green′ module were connected with metabolism, energy metabolism, and oxidative phosphorylation (Supplementary Table S4c).

To further study the molecular mechanism of CMS, the unigenes of the three modules were analyzed by GO enrichment and KEGG pathway. In GO analysis, the unigenes were mainly enriched in metabolic process (GO: 0030163), cellular process (GO: 0009765), cell (GO: 0005737), catalytic activity (GO: 0008171), and binding (GO: 0008270) (Supplementary Figure S1). In KEGG pathway analysis, the ′turquoise′ and ′blue′ modules were similar and mainly involved in translation (ko03010), carbohydrate metabolism (ko01200), folding, sorting, and degradation (ko04141), signal transduction (ko04075), and amino acid metabolism (ko01230) (Supplementary Figures S2 and S3). Genes in the ′green′ module were mainly related to energy metabolism (ko00910), carbon metabolism (ko01200), translation (ko03010), lipid metabolism (ko00071), and plant hormone signal transduction (ko040750) (Supplementary Figure S4).

### 2.7. Major Pathways and Genes Associated with CMS

It was found that numerous DEGs were involved in the metabolic pathways of male sterility in cotton C2P5A. Carbohydrate metabolism provides energy for anther development, four DEGs were involved in pentose and glucuronate interconversions (ko00040); 67 DEGs in starch and sucrose metabolism (ko00500); 42 DEGs in galactose metabolism (ko00052); 32 DEGs in fatty acid biosynthesis and metabolism (ko00061, ko01212); 17 DEGs in ascorbate and aldarate metabolism (ko00053); and 28 DEGs in glutathione metabolism (ko00480) (Supplementary Table S5a–f).

**Figure 8:**
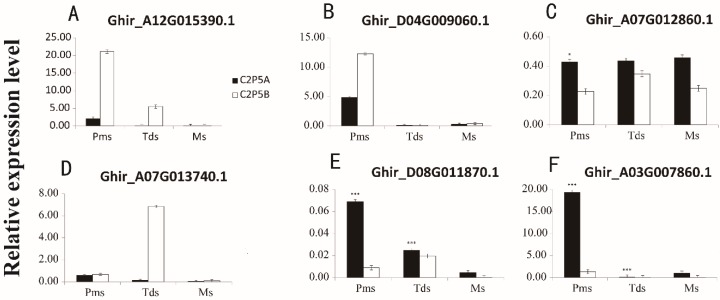
Expression patterns of DEGs in anther development. (**A**), (**B**), (**C**) and (**D**) The ROS detoxification related genes. (**E**) and (**F**) The tapetum development genes. PMs, pollen mother cell stage; Tds, tetrad stage; Ms, mononuclear stage. C2P5A, cytoplasmic male sterile line; C2P5B, maintainer line. The black columns indicate the male sterile line C2P5A, and the white columns indicate the maintainer line C2P5B. The error bars represent SD (Student′s *t*-test), *** *p* < 0.001, * *p* < 0.05, and absolute value of log_2_Ratio ≥ 2, *p* = 0 in (**C**), *p* = 0 in (**E**), *p* = 8.57921E−05 in (**F**), three biological replicates were performed.

A number of genes were associated with ROS detoxification. We identified 18 peroxisome (ko04146) genes encoding POD (peroxidase), CAT (catalase), and XOT (alternative oxidase) (Supplement Table S6a). In addition, nine aldehyde dehydrogenase family (ALDH) DEGs were identified (Supplement Table S6b). Of these genes, *Ghir_A12G015390.1* was down-regulated in three developmental stages (Figure 8A). Cytochrome c oxidase subunit VI mutations are associated with the male sterility of certain plants, we identified three associated DEGs (Supplement Table S6c). *Ghir_D04G009060.1* encoding cytochrome c oxidase subunit VI was down-regulated at Pms and Tds stage of anther development, then up-regulated at Ms stage (Figure 8B). We identified 13 DEGs which encoded cytochrome P450 (Supplement Table S6d). The expression of *Ghir_A07G012860.1* was up-regulated at three different stages of the C2P5A anther development (Figure 8C). We identified seven DEGs that encoded acyl-CoA synthetase (ACS1) (Supplement Table S6e) and the expression of *Ghir_A07G013740.1* was down-regulated at three different stages of the C2P5A anther development (Figure 8D).

Several genes associated with tapetum development were identified in the CMS line C2P5A. Two genes encoded the Jojoba acyl-CoA reductase-related male sterility protein (*Ghir_A08G000910.1, Ghir_D09G013360.1*). We identified nine up-regulated genes that encoded basic helix-loop-helix (bHLH) DNA-binding family proteins (Supplement Table S6f). Ghir_D08G011870.1 was up-regulated at three stages of C2P5A anther development (Figure 8E), the abnormal expression of these genes may be responsible for the male sterile line C2P5A. We identified 55 genes that were MYB domain proteins that regulate tapetum development (supplement Table S6g). *Ghir_A03G007860.1* was up-regulated at three stages of C2P5A anther development (Figure 8F).

### 2.8. Validation of DEGs by Quantitative Reverse-Transcription PCR (qRT-PCR)

qRT-PCR was used to verify the expression levels of DEGs identified. We identified seven genes associated with fertility at three different stages of anther development, choline/ethanolamine kinase (*Ghir_A10G019450.1*), ALDH involved in the glyoxylate cycle (*Ghir_A12G015390.1*), cytochrome c oxidase subunit VI (*Ghir_D04G009060.1*), cytochrome P450 (*Ghir_A07G012860.1*), ACS1(*Ghir_A07G013740.1*), and two transcription factors involved in tapetal development (*Ghir_D08G011870.1, Ghir_A03G007860.1*) (Table 2). Each gene underwent three biological replicates and each biological replicates was three times. Pearson’s correlation was used to evaluate the qRT-PCR and RNA-sequencing data. Overall, the expression levels of the seven DEGs were consistent with RNA-sequencing data (Supplementary Figure S5A; correlation coefficient = 0.68). The coefficients for Pms, Tds and Ms stage of anther development were 0.74, 0.83, 0.54, respectively (Supplementary Figure S5B–D). The results showed that our RNA-seq data were reliable and conducive to the identification of DEGs in anther development.

## 3. Discussion

The sequencing of several polyploid cotton plants has been completed, promoting the development of the cotton genome [69,73,74]. With these reference sequences, it was more convenient to use high-throughput sequencing technology to explore the molecular mechanism of CMS. RNA-sequencing techniques were used in cotton CMS-D8, CMS line H276A, CMS line zhong41A, and GMS line 1355A [25,27,61,75]. Except for the CMS line zhong41A, the selected samples are a mixture of anthers, ovaries and stigmas [27]. We selected samples similar to those in the CMS line zhong41A; flower buds of coincident length were selected, sepals and petals were peeled off, pure anthers were collected, and stigmas and ovaries were discarded. To obtain accurate and repeatable RNA-sequencing data, three biological replicates for each sample were used, and a high correlation coefficient (0.99–1) between replicates per sample were obtained, indicating sequencing results were credible and that the consistency of anthers collection was maintained.

### 3.1. The Crucial Period of Abortion in the CMS Line C2P5A

Normal development of anthers is required for the breeding and reproduction of flowering plants. Male sterility is a common phenomenon and often used to produce seeds by heterosis. It has been reported that flowering plants abort at different periods. In the photosensitive male sterility in cotton, there is no difference between the mutant and the wild type of anthers in pollen mother cells and tetrad stages. The tapetum of the mutant plant is degraded at the microspore stage, but the released microspore is empty and shriveled, so the abortion period of the photosensitive male sterile mutant occurs at the microspore stage [76]. In the GMS 1355A line, anther abortion occurs during the release of microspores (stage 8), microspores are shrunken and the exine lacks spines [25]. In CMS H267A, zhong41A male sterile lines, the pollen mother cell is gradually dissolved at the tetrad stage [27,75]. In their study, Rao and colleagues studied 36 male sterile lines from 14 cytoplasmic sources, but six without cytological material on male sterility. The results showed that in dicotyledonous plants, 27% of abortions occurred at the early stage of meiosis, 58% at the tetrad stage, and 15% at the microspore development stage [77].

In this research, C2P5A anthers do not form tetrads in the tetrad period, indicating that abortion may occur at the tetrad stage. According to previous reports, this period of abortion is called non-pollen male sterility [78], which is appropriate for studying the molecular mechanism of CMS.

### 3.2. Major Genes Regulate Anther Development in Cotton

Anther development is regulated by numerous genes, approximately 3500 genes are explicitly expressed in *Arabidopsis* anthers [79]. In the cotton (*G. hirsutum*) 21A GMS line and its maintainer line, 1742 genes were found to have differential expression [22]. 2446 DEGs were identified in the anthers of 1355AB line [25]. And a comparison of H276A and H276B anthers yielded 3603 DEGs [75]. In this study, we analyzed the transcript profiles of the cotton CMS line C2P5A and the maintainer line C2P5B. A total of 2013 genes were found to have differential expression by DEGseq. GO annotation and KEGG enriched pathway analysis showed that male sterility resulted in the differential expression of many genes.

CMS is associated with recombinant proteins encoded by mitochondria [58,80,81,82]. Aerobic respiration takes place in the mitochondrion and provides energy for various activities within the cell. The mitochondrion is the main source of ROS and thereby triggers or inhibits apoptosis [27,48,83].

ROS homeostasis is crucial for normal anther development [27]. A subtle ROS concentration is necessary for cell metabolism of various plant growth processes, such as root cell expansion and abiotic stress [27,84,85]. However, when there is an over-accumulation of ROS, which cannot be rapidly removed from cells, a ROS burst occurs and induces cell apoptosis and anther abortion [58,86]. MDA is an indicator of ROS in organisms and results from the enhancement of membrane lipid peroxidation, which damages the cell membrane and disturbs physiological metabolism inside the cell [87]. In the present study, MDA levels in C2P5A male sterile line were higher than those of the C2P5B maintainer line (Figure 3F). This proved that the C2P5A male sterile line accumulate excessive ROS during anther development. The results in cotton are consistent with U87B1-706A (wheat) [88] and 9704A (pepper) [87].

To better resist oxidative stress, plants form an efficient antioxidant defense system in the cell and reduce the impact of ROS on a variety of biological molecules, thus maintaining homeostasis [89]. SOD, POD, and CAT are necessary antioxidant enzymes that scavenge excess ROS [90]. In our study, at Pms stage of anther development and before anther abortion in C2P5A, a maximum increase of MDA content indicated excessive ROS accumulation, as the antioxidant defense system is activated, antioxidant POD and CAT levels increased (Figure 3D,E). Studies have shown that excessive accumulation of ROS may induce an antagonism oxidation system in biological organisms [58]. According to the enzymatic mechanism of ROS scavenging, we identified 18 peroxisome genes encoding POD, CAT, and XOT, at Pms stage of anther development. Seven genes are up-regulated and five down-regulated when ROS levels are highest.

The ALDH family plays an important role in mitochondria, which down-regulates and causes the cytotoxicity of excess acetaldehyde and ethanol in cells, contributes to ROS bursts and inhibits progress of the tricarboxylic acid cycle [91,92]. Nine genes encoding ALDHs were identified (Supplementary Table S6b). In previous study, cytochrome c oxidase subunit VI mutations were associated with male sterility in certain plants [75], for instance, in Beet G-CMS, cox2 expression decreased cytochrome c oxidase activity [93], and in the pepper CMS line, the cox2 gene was inserted into the orf456 chimeric gene [94].

Cytochrome c release and excessive ROS function are mostly retrograde signals that trigger plant PCD and lead to male sterility [95]. COX11 is the nuclear coding assembly factor of cytochrome c oxidase and is highly conserved in eukaryotes [96], for example, yeast COX11 (ScCOX11) [97] and rice COX11 (OsCOX11) [52]. In our research, three genes encoding cytochrome oxidase have been identified and C2P5A male sterility may be associated with them.

Cytochrome P450 is an oxidase involved in many biosynthetic pathways. In tapetum, fatty acid-CoA enters the endoplasmic reticulum and under the catalysis of cytochrome P450 family proteins performed hydroxylation. This process is essential for the formation of both cuticle and exine [98]. In this study, we identified 13 DEGs that encoded cytochrome P450. The expression of these genes in male sterile line was abnormal compared with the maintainer line, which may be related to pollen abortion in C2P5A.

Transcription factors are associated with anther development. The tapetum is an important structure for anther development. The tapetum and its function are regulated by numerous genes and transcription factors [99]. Basic helix-loop-helix (bHLH) domain transcription factors are mainly involved in the regulation of plant growth and development [100]. Most reported bHLH transcription factors related to tapetum cell and microspore development, abnormal function of bHLH transcription factors cause male sterility mutations, which often have similar characteristics and show precocious or delayed degradation of tapetum cells, resulting in blocked pollen formation and abortion. In *Arabidopsis*, the mutation that causes aborted microspores, DYT1, leads to the abnormal development of tapetum and premature degradation of microspores [40,101]. AtbHLH10, AtbHLH89, and AtbHLH91 interact with each other, and double or triple mutations show male sterility [47]. JAM1, JAM2, and JAM3, three bHLH transcription factors involved in jasmonic acid signal transduction, play a negative regulatory role of JA-mediated male sterility [102]. In rice, *UDT1, TDR, ETA1,* and *bHLH142/TIP2* encode bHLH transcription factors. The *UDT1* mutant has highly vacuolated tapetum cells during meiosis, the pollen mother cells do not divide into microspores, and there is no gradual degradation [103]. TDR, ETA1, and bHLH142/TIP2 regulate delayed tapetal degradation and aborted pollen formation [42,44,50,104]. OsbHLH138 regulates thermosensitive GMS in rice [105]. In cotton, Yang and colleagues identified four bHLH DEGs and two MYB130 DEGs that may be involved in abnormal tapetum development in zhong41A [27]. In this research, we identified nine up-regulated genes that coded for bHLH DNA-binding family proteins, the up-regulated expression of these genes may be responsible for male sterility of C2P5A.

MYB is an important transcription factor family that regulates anther development. AtMYB103 regulates the development of the tapetum and its abnormal expression causes male sterility [106]. AtMYB33 and AtMYB65 regulate the development of tapetum, and the double mutant has excessive vacuolation, swelling, and hypertrophy in tapetum cells [107]. *TDF1* encodes the R2R3 MYB transcription factor mostly involved in transcriptional regulation of tapetum cells and pollen mother cells [108]. *AtMYB21* mutant or overexpression of *AtMYB24* exhibit shorter anther filaments, delayed or no anther dehiscence, and fewer viable pollen grains [109]. Similarly, the GhMYB24 transcription factor is specifically expressed in pollen and involved in the regulation of late anther/pollen development [54]. In this study, 55 MYB transcription factors were identified. Their abnormal expression in male sterile line compared with maintainer line may be the cause of male sterility.

*ACS1* encodes acetyl-coenzyme A. In previous studies, it has been reported that ACS proteins may be localized in the outer membrane of pea chloroplasts and maturing safflower seeds, and its activity is associated with fatty acids and microsomes [110,111]. It was found that ACS proteins are essential for normal cuticle development and are involved in peroxisomal and fatty acid β-oxidation in *Arabidopsis* [22,112,113,114]. However, *GHACS1* gene plays an important role in the formation of microspores in the early anther development of cotton and is highly expressed in sporogenous cells, pollen mother cells, microspores, and tapetum cells. Inhibition of *GHACS1* seriously affected the development of tapetum cells and the formation of normal microspores [53]. In cotton anthers, the genes were involved in fatty acid metabolism and peroxisome. Abnormal expression was identified in two genes in the male sterile line, which encoded Jojoba acyl-CoA reductase-related male sterility protein.

### 3.3. Major Metabolic Pathways Regulating Anther Development in Cotton

Carbohydrate metabolism, which provides carbon sources and energy for plant growth and development, is the most basic pathway of plant metabolism [115,116]. Carbohydrates provide energy and nutrients for anther development. The period of anther and pollen development are intensively energy-demanding [81,117]. Perturbed carbohydrate metabolism can significantly damage pollen development and cause male sterility [118,119].

It was recently reported that glycolysis was activated in the anthers of 1355A, which caused a decrease of soluble sugars, such as fructose and glucose, and accumulation of acetyl-CoA, which led to significant increases of c14:0 and c18:1 free fatty acids. The genes involved in fatty acid synthesis are crucial to regulate normal pollen hydration and plant fertility. High rates of glucose metabolism may promote fatty acid synthesis to enable anther growth [120].

Previous studies have shown that 1165 genes associated with flavonoid and ascorbate-glutathione cycle, starch and sucrose metabolism are important during anther development in cotton [19]. Using cotton anthers from wild type and GMS lines analyzed by digital gene expression (DGE), identified many of the essential genes required for cotton anther development. The genes were mainly associated with sucrose and starch metabolism, the pentose phosphate pathway, glycolysis, and flavonoid metabolism [20].

Some researchers identified 1742 significant DEGs between anthers of GMS line (B) and its maintainer line (K) in cotton plants by the DGE approach showed that sugar metabolism such as the interconversion of pentose and glucuronate, starch and sucrose metabolism, galactose metabolism were required for the development of cotton anthers [22].

In the present study, we identified 2013 genes that were differentially expressed between the male sterile line C2P5A and the maintainer line C2P5B using a DEG seq approach. These DEGs were analyzed by GO enrichment and KEGG pathways and were found to be associated with starch and sucrose metabolism, galactose metabolism, ascorbate and aldarate metabolism, glutathione metabolism, and pyruvate metabolism, pentose and glucuronate interconversions, and fatty acid biosynthesis and metabolism.

Proline interacts with carbohydrates to provide nutrients, promoting pollen development and pollen tube elongation. Proline also accompanies the development of microspores [121].

Soluble proteins contain enzymes involved in plant growth and development which play crucial roles in the development of anthers and microspores. However, soluble protein in the anther of male sterile line were deficient, resulting in abnormal protein synthesis in the anther [122].

In our research, biochemical determination of soluble sugar and soluble protein content of the CMS line C2P5A was lower than that of the maintainer line C2P5B at the three stages of anther development (Figure 3A–C), which as well as, lack of energy and nutrition may lead to pollen abortion in C2P5A.

## 4. Materials and Methods

### 4.1. Plant Materials

The cotton (*Gossypium hirsutum* L.) plants C2P5A (CMS line) and C2P5B (maintainer line) used in these experiments were cultivated in the experimental field in Henan Institute of Science and Technology, Henan, China. 2018–2019, April 27 sowing, line spacing 1 meter, plant spacing 40 centimeter, field watering was irrigated at the seedling stage, flowering stage and flower bud stage respectively. Organic fertilizer and compound fertilizers were used as field base fertilizers. During anthesis, both lines were identified according to flower apparatus morphology, the anther vigor with 2% TTC staining, and anther development stage with 1.5% (*w*/*v*) acetocarmine staining. Plant lines were photographed under a microscope and SEM (Epson Expression 12000XL, Nagoya, Japan).

Flower buds had lengths of 3–4 mm (pollen mother cell stage), 4.1–5.0 mm (tetrad stage), 5.1–6.0 mm (mononuclear stage), and flower bud length (length nectary to bud apex, mm). Anthers were collected from three different periods of the CMS line and maintainer line. Sepals and petals were peeled off, pure anthers were collected, and stigmas and ovaries were discarded. The anthers were carefully and quickly isolated and frozen in liquid nitrogen and stored at −80 °C until use.

### 4.2. Histological Analysis

To identify the anther abortion period of the male sterile line C2P5A, flower buds of different periods were collected and fixed in FAA [10% formalin, 5% acetic acid, and 50% ethanol (*v*/*v*)].

Samples were sectioned in paraffin and fixed, washed, dyed-saffron, dehydrated, hyalinized, infiltrated, embedded, sectioned, exhibited, re-dyed-solid green, and sealed according to a previous report [75].

### 4.3. Measurement of Physiological Indices

Buds were collected from three different periods of the CMS line C2P5A and the maintainer line C2P5B, and sepals and petals were removed. Soluble sugar was measured by anthrone [123], pure glucose was used as standard; Soluble protein was measured according to Lowry et al. [124], bovine serum albumin (BSA) was used as standard. Determination of MDA content was by thiobarbituric acid-reactive-substances (TBARS) assay [125], antioxidative enzymes (POD, CAT) were measured using quantificationally by ELISA instrument (TECAN, Tech Spark 10M, Zurich, Switzerland) [126]. There were three biological replicates per sample.

### 4.4. Screening of DEGs and Co-expression Network Analysis of Unigenes

Total RNA was isolated from harvested anthers using an RNAprep pure PlantKit (Tiangen, Beijing, China); with three biological replicates per sample. The RNA concentration was assessed by NanoDrop (Waltham, MA, USA). The RNA quality was tested using an Agilent 2100 instrument (Santa Clara, CA, USA) with 28S/18S rRNA ratio > 1.6 (Agilent 2100) per sample for library construction on the BGISEQ-500 platform (BGI, Wuhan, China). SOAP [127], the short oligonucleotide alignment tool, matched raw reads to the ribosome database, a maximum of five mismatches were allowed. Reads with low-quality rRNA, containing adapters, joint contamination, and high levels of unknown bases (“N”) were filtered to produce clean reads. Clean reads were matched to the reference genome (http://cotton.hzau.edu.cn/EN/download.php) and Cotton Gene database (https://www.cottongen.org/) by HISAT [69,128]. The transcriptional assembly was performed by StringTie [129] and Bowtie2 [67]. FPKM was used to calculate gene expression levels by RSEM [68]. DEGs were identified using DEGseq [70] in accordance with the threshold values: |log_2_Ratio| ≥ 2, readnum >3, *p* ≤ 0.001 and the criteria of FDR < 0.01. GO annotation and KEGG pathway analyses were performed for the DEGs.

The correlation network of the WGCNA R package is often used for correlation analysis between highly correlated DEG modules and samples to find modules of high specific expression in samples [130]. Module Eigengenes (ME) values were used to estimate associations between modules and the CMS phenotype.

### 4.5. RT-PCR Analysis for Gene Expression

Total RNA was extracted using an RNA prep pure PlantKit (Aidlab Biotech, Beijing, China), according to the manufacturer′s instructions. RNA was reverse transcribed using a TransScript^®^ One-Step gDNA Removal kit and a cDNA Synthesis SuperMix kit (TransGen, Beijing, China). Quantification PCR (qRT-PCR) was used to confirm the RNA-seq data. Genes related to C2P5A male sterility of the transcriptome were selected. The specific primers for RT-PCR were designed using Primer Premier 6.0 software (http://www.premierbiosoft.com/crm/jsp/com/pbi/crm/clientside/ProductList.jsp) and synthesized by Sangon Biotech (Shanghai, China) (Supplementary Table S7a,b). The reactions for RT-PCR were performed using cDNA as a template, using a qPCR SuperMix Kit (TransGen, Beijing, China). Each reaction was performed in three biological and three technical replicates on a QuantStudio 6 Flex instrument (Applied Biosystems, Foster city, CA, United States). RT-PCR analysis was performed according to the protocol of the TransStart^®^ Top Green qPCR SuperMix with Two-Step kit (TransGen, Beijing, China).

The PCR circulation conditions included denaturation at 95 °C for 30 s, followed by 45 cycles of denaturation at 94 °C for 5 s, annealing and extension at 60 °C for 30 s. The melting curve was determined for each sample. Relative expression was calculated for every sample using the cycle threshold (Ct)2^−ΔΔCt^ method [131].

## 5. Conclusions

In this study, we compared the cytology, the physiological characteristics and the transcriptome of the cotton CMS line C2P5A and its maintainer line C2P5B. Cytology and physiological characteristics results indicated that pollen abortion in C2P5A occurred at the tetrad stage. The transcriptome results revealed 2013 DEGs between C2P5A and C2P5B. Bioinformatic analyses showed that DEGs were mainly related to encoding ROS detoxification enzymes, tapetum proteins or transcription factors, vital metabolic pathways including starch and sucrose metabolism, galactose metabolism, ascorbate and aldarate metabolism, glutathione metabolism, and pyruvate metabolism. This research results provide a basic theory and molecular mechanism for CMS research and will accelerate research on CMS in cotton.

## Figures and Tables

**Table 1:** Summary of tag numbers.

Sample	Raw Data	Clean Data	GC (%)	Q30(%)	(%)of Total Mapped	Total Gene Number	Total Isoforms	Novel Isoforms
MsPms1	96339898	84112026	44.33	97.65	77.42	85012	126455	49311
MsPms2	96339060	84728466	44.43	97.84	75.51	83758	122693	48159
MsPms3	96340692	84822750	44.52	97.81	75.44	85170	125779	49293
MsTds1	66418638	60016542	44.22	98.33	76.38	83808	122636	47699
MsTds2	96341884	84562392	43.57	97.68	77.36	86647	129005	50208
MsTds3	96340878	84830410	44	97.77	74.54	86626	126787	50096
MsMs1	70226770	62112146	45.21	98.35	78.11	82246	117948	45803
MsMs2	96340984	84269862	44.5	97.81	77.01	85808	124373	49192
MsMs3	75363530	68729552	44.43	98.45	79.47	85289	123996	48513
MPms1	78357630	71140510	43.98	98.42	77.1	84009	123651	49187
MPms2	93592588	83300884	43.81	97.97	76.95	84628	124284	49724
MPms3	94122576	81326894	44.24	97.83	73.56	84969	124580	49711
MTds1	93071774	84922682	43.79	98.38	78.87	84527	124186	49781
MTds2	79502370	72818484	43.85	98.23	80.11	83170	121809	48480
MTds3	71210292	64700084	43.83	98.33	79.25	82748	120584	48031
MMs1	96340778	84992800	44.36	98.03	75.37	84192	124176	48798
MMs2	96340324	84734142	43.65	97.83	77.69	84433	124979	49064
MMs3	74569750	67998900	43.6	98.23	75.57	82250	120841	47466

MS, male sterile line C2P5A; M, maintainer line C2P5B; 1, 2, 3 represent the biological duplications; Pms, pollen mother cell stage; Tds, tetrad stage; Ms, mononuclear stage.

**Table 2:** qRT-PCR confirmation of DEG expression profiles.

Gene ID	Protein Identity	Pms(Fold Change)		Tds(Fold Change)		Ms(Fold Change)	
		qRT-PCR	RNA-seq	qRT-PCR	RNA-seq	qRT-PCR	RNA-seq
Ghir_A10G019450.1	Cell wall/membrane/envelope biogenesis	−5.41	−2.68	4.51	3.15	3.47	2.37
Ghir_A07G013740.1	Long chain acyl-CoA synthetase	−1.59	−2.56	−3.52	−2.64	−0.16	−2.88
Ghir_A12G015390.1	Aldehyde dehydrogenase family	−3.88	−6.73	−6.10	−9.37	−1.05	−6.86
Ghir_D08G011870.1	bHLH-MYC transcription factors	2.87	2.13	2.00	2.16	4.67	2.52
Ghir_A07G012860.1	Cytochrome P450	0.98	2.12	0.23	2.50	0.98	2.44
Ghir_D04G009060.1	Cytochrome oxidase c subunit VIb	−4.53	−3.49	−0.29	−2.37	1.87	3.11
Ghir_A03G007860.1	Myb-like DNA-binding domain	1.61	2.56	3.70	2.23	5.73	2.19

Fold change refers to the expression in the male sterile line C2P5A when compared to the maintainer line C2P5B. A negative and positive value refers to down- and up-regulation, respectively in the male sterile line C2P5A.

## Supplementary Materials

Supplementary materials can be found at https://www.mdpi.com/1422-0067/20/20/5120/s1.

## Abbreviations

CMS	Cytoplasmic male sterility
GMS	Genic male sterility
MS	Male sterility
DEG	Differentially expressed genes
GO	Gene ontology
KEGG	Kyoto Encyclopedia of Genes and Genomes
RSEM	RNA-Seq by Expectation Maximization
RNA-seq	RNA-sequencing
qRT-PCR	Quantitative Reverse-Transcription PCR
DGE	Digital gene expression
WGCNA	Weighted correlation network
TTC	Triphenyltetrazolium chloride
SEM	Scanning electron microscopy
PCD	Programmed cell death
