# Supplementary figures and images for: Using Transcriptome Analysis to Screen for Key Genes and Pathways Related to Cytoplasmic Male Sterility in Cotton (Gossypium hirsutum L.)
Source: Int J Mol Sci. 2019 Oct 16;20(20):5120. doi: 10.3390/ijms20205120 (PMC6830320; doi:10.3390/ijms20205120)

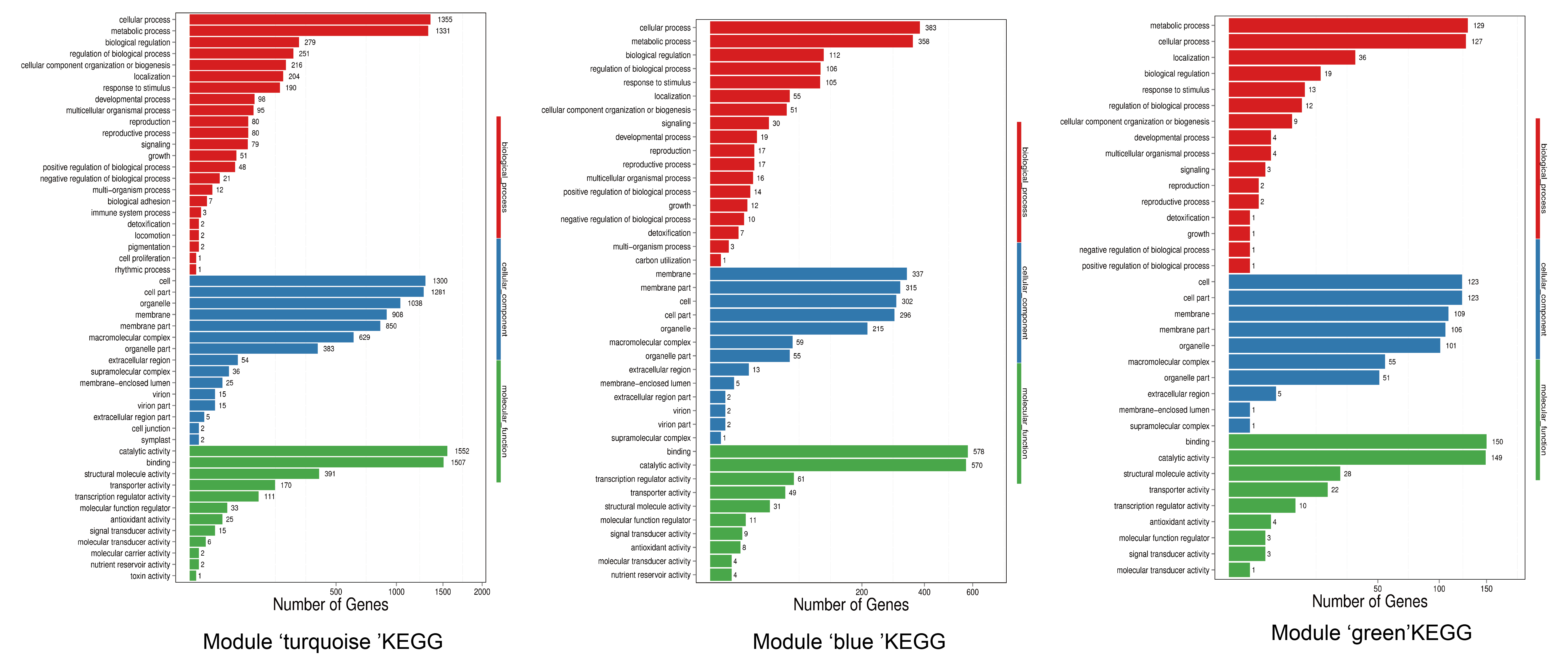

Supplement: Supplementary file 1 [file ijms-20-05120-s001.zip › ijms-602343-final-supplymentary/Supplementary Figure S1. module eigengenes genes GO enrichment.png]

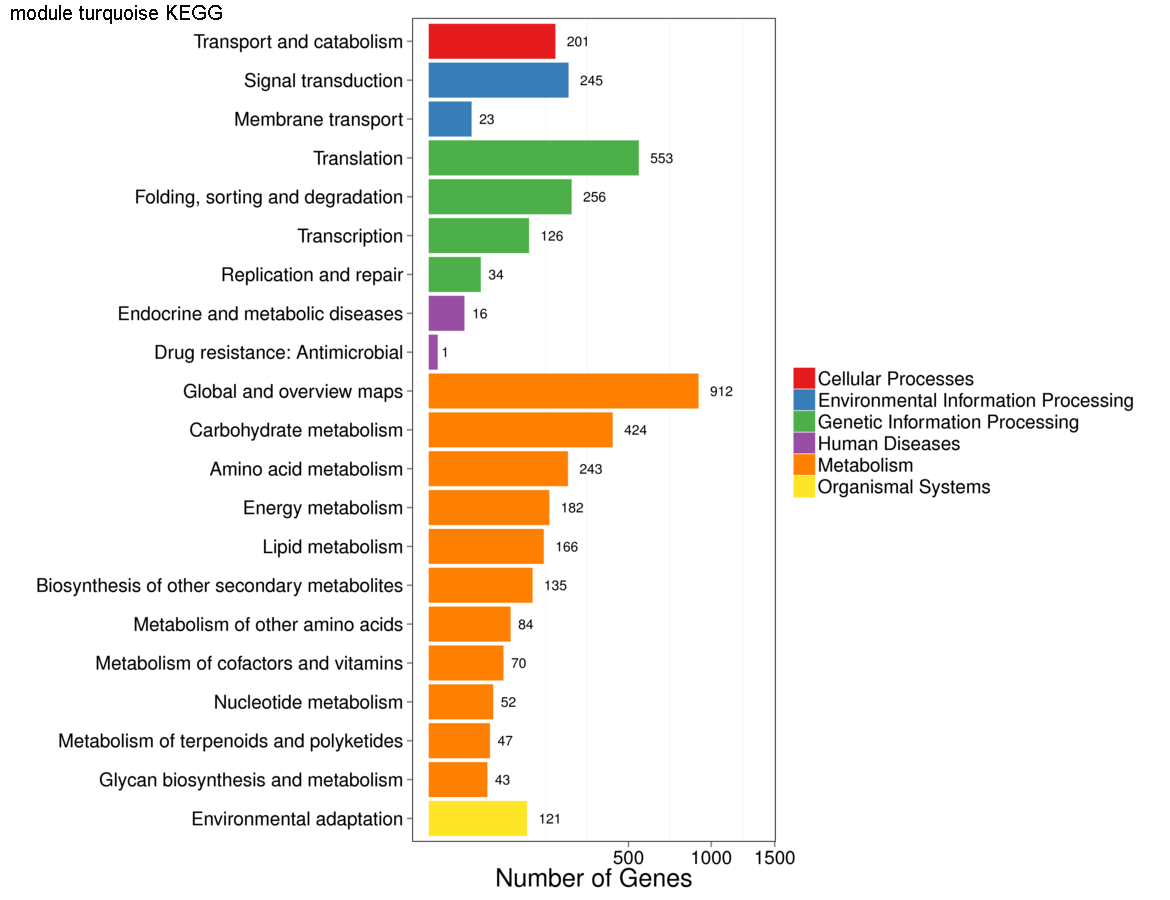

Supplement: Supplementary file 1 [file ijms-20-05120-s001.zip › ijms-602343-final-supplymentary/Supplementary Figure S2.module turquoise.KEGG.tif]

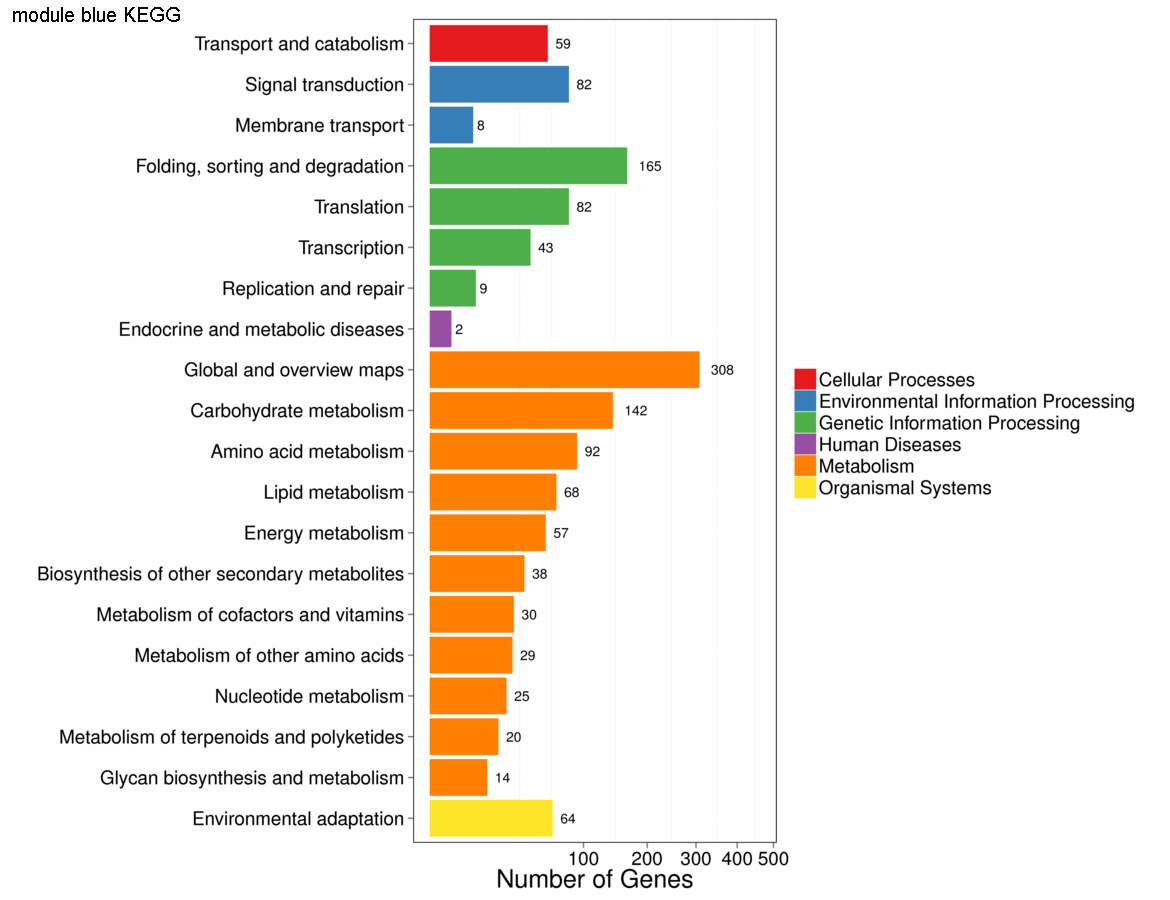

Supplement: Supplementary file 1 [file ijms-20-05120-s001.zip › ijms-602343-final-supplymentary/Supplementary Figure S3.module blue.KEGG.tif]

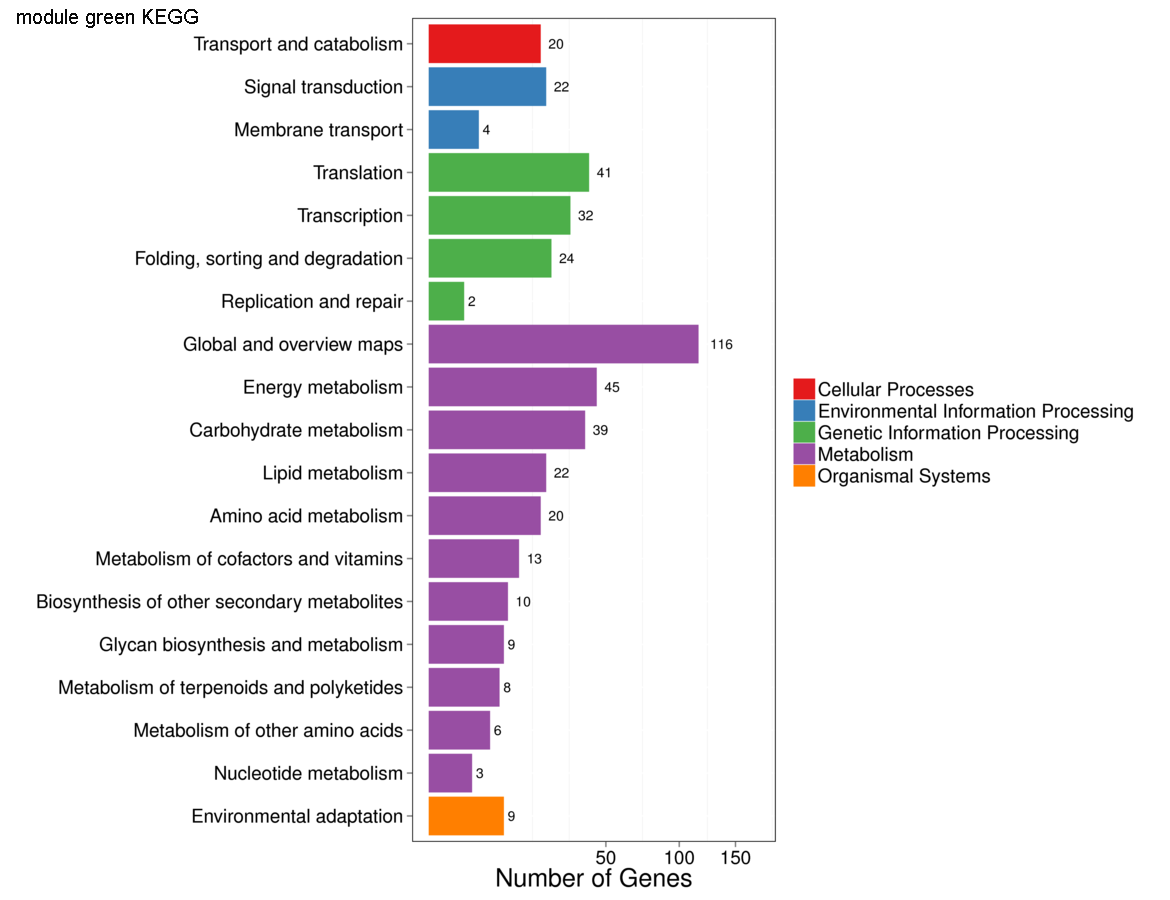

Supplement: Supplementary file 1 [file ijms-20-05120-s001.zip › ijms-602343-final-supplymentary/Supplementary Figure S4.module green.KEGG.tif]

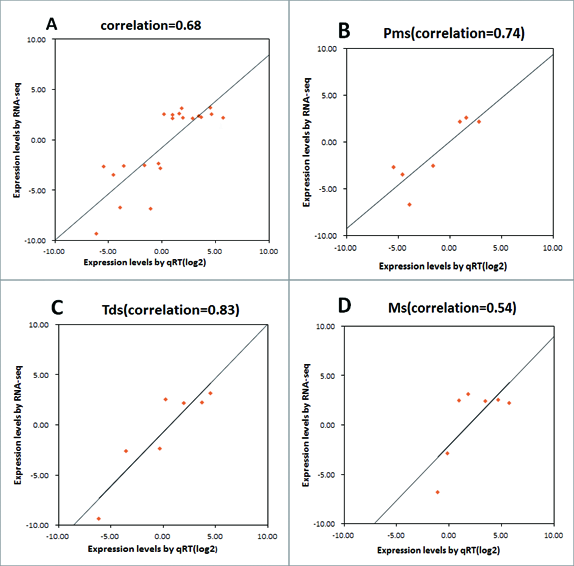

Supplement: Supplementary file 1 [file ijms-20-05120-s001.zip › ijms-602343-final-supplymentary/Supplementary Figure S5A-D.RT-PCR corrlation.tif]
